# Phenotype analysis of cultivation processes via unsupervised machine learning: Demonstration for *Clostridium pasteurianum*

**DOI:** 10.1002/elsc.202100114

**Published:** 2021-12-10

**Authors:** Yaeseong Hong, Tom Nguyen, Philipp Arbter, Tyll Utesch, An‐Ping Zeng

**Affiliations:** ^1^ Institute of Bioprocess and Biosystems Engineering Hamburg University of Technology TUHH Hamburg Germany

**Keywords:** automated fermentation analysis, *Clostridium pasteurianum*, phenotype analysis, process monitoring, unsupervised learning

## Abstract

A novel approach of phenotype analysis of fermentation‐based bioprocesses based on unsupervised learning (clustering) is presented. As a prior identification of phenotypes and conditional interrelations is desired to control fermentation performance, an automated learning method to output reference phenotypes (defined as vector of biomass‐specific rates) was developed and the necessary computing process and parameters were assessed. For its demonstration, time series data of 90 *Clostridium pasteurianum* cultivations were used which feature a broad spectrum of solventogenic and acidogenic phenotypes, while 14 clusters of phenotypic manifestations were identified. The analysis of reference phenotypes showed distinct differences, where potential conditionalities were exemplary isolated. Further, cluster‐based balancing of carbon and ATP or the use of reference phenotypes as indicator for bioprocess monitoring were demonstrated to highlight the perks of this approach. Overall, such analysis depends strongly on the quality of the data and experimental validations will be required before conclusions. However, the automated, streamlined and abstracted approach diminishes the need of individual evaluation of all noisy dataset and showed promising results, which could be transferred to strains with comparably wide‐ranging phenotypic manifestations or as indicators for repeated bioprocesses with clearly defined target.

**Abbreviations**
BES: bioelectrochemical system
CD: cosine distance
DBSCAN: density‐based spatial clustering of applications with noise
PCHIP: Piecewise Cubic Hermite Interpolating Polynomial
SED: squared Euclidean distance

## 1 INTRODUCTION

Machine learning applications are rapidly expanding throughout multiple research areas with promising opportunities [1, 2, 3]. In the field of life sciences, several new applications were developed in the past decades on multiple omics levels, such as genome analysis [4, 5], classification of transcriptomics and epigenetic data [6, 7], protein‐protein interaction or protein engineering [8, 9] and data analysis for metabolomics [10, 11]. Further, prediction methods as support for successive work processes (e.g. protein engineering via directed evolution or strain engineering) can efficiently accelerate the practice [12, 13]. For fermentation‐based production of bulk chemicals and other high‐value compounds, the fermentation process constitutes a unit operation for a specific chemical conversion or biochemical production that utilizes microorganisms as microbial cell factory. Providing and maintaining the most effective and efficient fermentation conditions, which enable or trigger specific phenotypic behavior, is one of the key tasks of fermentation control for yield and productivity maximization [14, 15, 16]. In this regard, several previous studies employing machine learning were reported, which employed artificial neural network for fed‐batch fermentation of iturin A [17], for fermentation of wheat germ producing anti‐tumor benzoquinones [18], for bioethanol production via *Saccharomyces cerevisiae* fermentation [19] and for optimization of xylitol production bioreactor parameters [20]. Prediction of optimal temperature as physical condition via machine learning was demonstrated by Li et al. [21].

**PRACTICAL APPLICATION**

Identification of phenotypic manifestation for bioprocesses constitutes an essential element to characterize cellular behavior and monitor fermentation processes. Here, we demonstrate a unsupervised learning method for automated processing of time series of raw concentration data from multiple cultivations of the bacterium *Clostridium pasteurianum* in order to identify, cluster and output distinct reference phenotypes. In contrast to individual evaluation of single cultivation experiment, manual assessment is no longer required, which also avoids any risk of subjectively influencing and overlooking potential candidates. The resulting reference phenotypes can be used for streamlined phenotypic examinations in a manageable manner, since exceptionally high number of data are abstracted to most relevant and distinctive reference phenotypes. Especially for strains such as *C. pasteurianum*, with highly varying phenotypic manifestations or for repeating fermentation processes with clearly defined phenotypic manifestation, the capture of phenotypes and its use as references and indicators are the assets of this method.

In this work, a machine learning‐based method is presented for analysis of fermentation‐based bioprocesses by clustering cellular manifestation. Its fundamental idea arose from subjective impressions during manual analysis of multiple cultivation experiments to identify and quantify phenotypic behaviors: the anaerobe bacterium of interest, *Clostridium pasteurianum*, exhibits strong variations of phenotypic behavior in solventogenesis and acidogenesis. In order to detect and to quantify “generic” phenotypic manifestations (as collective of cellular behavior), single cultivation experiments were analyzed and sorted by hand. However, difficulties arise, when multiple cultivations are taken into account due to differences and dynamics of cultivation conditions and cellular response. Depending on the number of experiments, manual sorting of cultivation data into specific categories of phenotypic manifestations can be tedious and carries the risk to subjectively influence and to overlook potential candidates. Based on re‐appearance of typical phenotypic expressions throughout multiple cultivation experiments, the question raises, if an automated method could be introduced that “learns” from an entirety of (noisy) raw sampling data and simply outputs reference phenotypic manifestations. The reference manifestations are then to be employed as manageable, unique and simplified abstractions of cellular behavior.

Employing unsupervised learning method as a field of machine learning, patterns can be identified by grouping data points into meaningful clusters, which requires only input data for the algorithm and data points are not manually divided into categories [22]. To establish a common basis of calculative quantities, the phenotypic manifestation was defined as a set of biomass‐specific rates (e.g. growth rate, specific consumption or production rate) forming a vector. These rates are to be calculated from all cultivation experiments and vectors are then to be clustered based on the underlying hypotheses: (a) the time‐dependent entirety of a cellular behavior can be sufficiently represented as a vector consisting of biomass‐specific rates; (b) grouped (clustered) “phenotypic behaviors” can be represented by a single centroid that is approximated as medians of all dimensions. Here, the necessary computing process, used parameters and the employed strategies are described. Further, additional examples of phenotype analysis are provided that employ identified clusters for *C. pasteurianum*, demonstrating the perks of this method, while the potentials and limitations are discussed.

## 2 MATERIALS AND METHODS

### 2.1 Strains, cultivation, and analytics

All used data sets of cultivation data can be found in Mendeley Data repository. For this work, cultivation data of different *Clostridium pasteurianum* strains (Supporting Information 2) were used with cultivation and analytical methods as described previously [23, 24, 25, 26]. Briefly, stock cultures were stored as 20% v/v glycerol stocks at ‐80°C. Pre‐culture was grown in Reinforced Clostridia Medium (RCM) or 2 × YTG medium and inoculated to modified Biebl medium, which was adapted from Biebl [27], with additional additives (glycerol, glucose, yeast extract, CaCO_3_, FeSO_4_∙7H_2_O, l‐cysteine∙HCl∙H_2_O, sodium formate, biotin, neutral red, brilliant blue) for cultivation experiments. For the plasmid harboring mutants (PC and GCSY1), thiamphenicol was supplemented between 7 to 14 μg mL^−1^. Fermentations were carried out in 2 L foil or glass reactors (Bioengineering AG, Wald, Switzerland), 1.5 L or 300 mL DASGIP Parallel Bioreactor Systems (DASGIP Eppendorf, Jülich, Germany) and bioelectrochemical fermentations were conducted with the AIO electrode [28]. For small‐scale anaerobic cultivations without pH‐control, 100 or 200 mL serum bottles were employed, in which multiple samples over cultivation time were drawn. Concentrations of substrates (glycerol and glucose) and extracellular metabolites (1,3‐propanediol, ethanol, butanol, acetate, butyrate, lactate and formate) were quantified using HPLC as described by Sabra et al. [29]. Biomass concentration was determined turbidometrically at 600 nm [30].

### 2.2 Cluster formation

For the calculation of specific rates and cluster formation, MATLAB 2020b (MathWorks, Natick, MA, USA) was used and the script can be found in Mendeley Data repository, which is schematically depicted in Supporting Information 1. Inter‐sample concentrations $c_{D}\left(t_{j}\right)$ (with D=1,2,…m representing biomass, substrate and products) were approximated using PCHIP‐function [31] as previously described in [26]. For the calculation of biomass‐specific rates $r_{D}\left(t_{j}\right)$ [mmol g^‐1^ h ^‐1^] (1), linear slope was approximated as time derivative of $c_{D}$ for each compound D. In case of biomass (D=1), specific production rate corresponds to the growth rate $\mu (t_{j})$ [h^−1^] and was calculated via exponential fit (2).

(1) $$r_{D,t}=\frac{1}{c_{1}\left(t\right)}{\left.\frac{dc_{D}\left(t\right)}{\mathrm{dt}}\right|}_{t}\approx \frac{1}{c_{1}\left(t\right)}\frac{\Delta c_{D}}{\Delta t}\;$$

(2) $$\mu_{t}=r_{1,t}=\frac{1}{c_{1}\left(t\right)}\frac{dc_{1}\left(t\right)}{dt}$$

This calculation of rates using concentrations from samplings was performed for all datasets of cultivation experiments $ce_{p}\;$($p\;=\;1,2,\text{…}p_{max}\;$representing each cultivation experiment). From all calculated rates $r_{D}$, outlier removal of 3th and 97th percentiles for each compound D was performed to dampen potential cluster misalignments due to calculated rates that are sensitive at low or high biomass concentrations except for Density‐based spatial clustering of applications with noise (DBSCAN) clustering. The generated dataset contains for each $t_{j}$ a specific set of μ and $r_{D}$ of different compounds, which can be depicted as a vector $a_{j,p}={\left(\mu \;r_{D=2}\;r_{D=3}\text{…}r_{D=m}\right)}^{\prime }\;{|}_{t=t_{j},\;ce=ce_{p}}\;$ that describes a specific biological phenotype found at cultivation time $t_{j}$ of the cultivation p.

Then, the datasets were normalized to *z*‐scores (3) with S as sample standard deviation (4) for each compound D=[1,m] to avoid scalar‐based weighting of specific elements, yielding in $\alpha_{j,p}^{*}={\left(z_{j,p_{1}}\;z_{j,p_{2}}\text{…}\;z_{j,p_{m}}\right)}^{\prime }\;$. For identification of patterns or clusters of all vectors, the following unsupervised learning methods were applied: k‐means clustering [32, 33] and DBSCAN [34]. Briefly, the centroid‐based clustering algorithm, k‐means clustering, iterates the position of the centroids ζ for the given number of clusters k with the objective of minimizing the sum of all point‐to‐cluster‐centroid distances. In addition, k‐means++ algorithm [32] was applied for center initialization for replicates. The following distance metrics were used for the distance calculation: squared Euclidean distance (SED) (5) and cosine distance (CD) (6). For k‐means clustering, different clustering evaluation methods can be applied to estimate the optimal number of clusters k. In this work, evaluations using the Gap criterion [35] and the Silhouette criterion [36] with SED and CD were applied with manual upper limit of 30 without repetition. In addition, the Davies‐Bouldin criterion [37] and the Calinski‐Harabasz criterion [38] were used with SED. The density‐based clustering method sorts all observations (vector $\alpha_{j,p}^{*}$) into core, border or noise points fulfilling the criteria of ε (scalar for neighborhood search radius for each $\alpha_{j,p}^{*}$) and $n_{p_{min}}$ (minimum number of neighbors for a core point). Found core points matching the criteria of ε and $n_{p_{min}}$ correspond to a cluster. DBSCAN‐parameters (ε, $n_{p_{min}}$) were manually screened. The formed clusters from the described methods were separately analyzed and characterized. For that, re‐scaled centroid ζ was estimated as medians of all $a_{j,p}$ of the same cluster.

(3) $$z_{j,p_{D}}=\frac{\left(a_{j,p_{D}}-\bar{a_{j,p_{D}}}\right)}{S_{D}}=a_{j,p_{D}}^{*}$$

(4) $$S_{D}=\sqrt{\frac{\sum_{j=1,p=1}^{j=j_{\max},\;p=p_{\max}}{\left(a_{j,p_{D}}-\bar{a_{j,p_{D}}}\right)}^{2}}{n-1}}$$

(5) $$\begin{array}{c} d_{\mathrm{SED}}\left(\alpha_{j,p}^{*},\zeta^{*}\right)={\left\|\alpha_{j,p}^{*}-\zeta^{*}\right\|}_{2}^{2}=\sum_{D=1}^{m}{\left(\alpha_{j,p_{D}}^{*}-\zeta_{D}^{*}\right)}^{2} \end{array}$$

(6) $$\begin{array}{ccc} d_{\mathrm{CD}}\left(\alpha_{j,p}*,\zeta *\right) & = & 1-\cos\left(\theta \right)=1-\frac{a_{j,p}^{*}\cdot \zeta^{*}}{{\left\|a_{j,p}^{*}\right\|}_{2}{\left\|\zeta^{*}\right\|}_{2}}\\ & = & 1-\frac{\sum_{D=1}^{m}a_{j,p_{D}}^{*}\zeta_{D}^{*}}{\sqrt{\sum_{D=1}^{m}{a_{j,p_{D}}^{*}}^{2}}\sqrt{\sum_{D=1}^{m}{\zeta_{D}^{*}}^{2}}} \end{array}$$

### 2.3 Analysis of clusters

To compare generated clusters in a radar plots, centroids of z of each cluster were re‐scaled for each element D=[1,m] from 0 to 1. For specific rates of substrates (negative values representing consumption of the glycerol and/or glucose), the signs were changed and labeled as consumption to improve the comparability. To detect potential conditional correlations of cluster prevalence related to datasets, which were not directly included for cluster formation and assignment (e.g. concentration ranges of substrate, product, cultivation condition), the logarithmic deviation of proportions between a specific cluster CL =1,2,…krepresenting cluster number and the total dataset for a given condition cond was calculated as in (7), where $n_{CL,\alpha_{j,p}}$ equates to the number of $\alpha_{j,p}$ assigned to the cluster CL. The sample population consists of all $a_{j,p}$ that are included in the cluster generation. Since sampled glycerol concentrations from fed‐batch fermentations were entered as accumulated amount of consumed glycerol, these sample points were excluded for the analysis for conditionality based on concentrations of substrates and metabolites. Otherwise, concentrations of substrates and metabolites were rounded in 1, 5, 10, 20, 50, and 100 mmol L^‐1^ steps for pooling as concentration ranges.

(7) $$\delta_{cond}\left(CL\right)=\log_{10}\left(\frac{\frac{{\left.n_{CL,\alpha_{j,p}}\right\rfloor }_{cond}}{n_{CL,\alpha_{j,p}}}}{\frac{\sum_{CL=1}^{k}{\left.n_{CL,\alpha_{j,p}}\right\rfloor }_{cond}}{\sum_{CL=1}^{k}n_{CL,\alpha_{j,p}}}}\right)$$

### 2.4 Superposition of cluster centroids

The utilized MATLAB script can be found in Mendeley Data repository. As iterative approach of depiction of $a_{j,p}$ (specific phenotypic state, e.g. steady state phenotype from a continuous fermentation) or series of $a_{j,p}$ (dynamic phenotype behavior of culture broth, e.g. phenotypes of a batch fermentation) superposition principle was applied. Under the assumption that the cellular (phenotypic) behavior can be approximated as superposition of cluster centroids with a certain distribution, the non‐negative least squares fitting problem (8) was solved, where $A^{**}$ (9) equates a m×k matrix for m and k as total number of compounds and clusters, respectively. To avoid weighting due to varying scales of the elements of D=[1,m] in $A^{**}$, centroids $\zeta_{D,CL}$ and $a_{j,p}$ were re‐scaled for each D from 0 to 1, resulting in $\zeta_{D,CL}^{**}$ and $a_{j,p}^{**}$. As indicator for quality of the fitting, the residual term $y^{**}$ (10) was used to calculate the residual sum of squares (RSS) (11) for all elements of $y^{**}$. Smallest residual sum of squares speaks in favor for a good approximation of data points via the combination of clusters and represents the best possible solution. To describe the fittings via $x^{**}$ (vector of variables, $x_{CL}^{**}$ with CL=[1,k], for the non‐negative least squares fitting problem) as distributions of clusters, the proportion $x_{j,p,CL}$ for cluster CL fitting the phenotype found at $t_{j}$ from the experiment $ce_{p}$ was calculated as in (12). For superposition approximation of time‐series data (e.g. batch fermentation), Gaussian‐weighted moving average was calculated over a window of five vectors to smooth time‐dependent fluctuations.

(8) $$\min_{x^{**}}{\left\|A^{**}\cdot x^{**}-a_{j,p}^{**}\right\|}_{2}^{2},x^{**}\geq 0$$

(9) $$A^{**}=\left(\begin{array}{cccc} \zeta_{D=1,CL=1\;}^{**} & \zeta_{D=1,CL=2\;}^{**} & \cdots & \zeta_{D=1,CL=k\;}^{**}\\ \zeta_{D=2,CL=1\;}^{**} & \zeta_{D=2,CL=2\;}^{**} & \;\cdots & \zeta_{D=2,CL=k\;}^{**}\;\\ \vdots & \;\vdots & \;\ddots & \vdots \;\\ \zeta_{D=m,CL=1\;}^{**} & \zeta_{D=m,CL=2\;}^{**}\; & \;\cdots & \zeta_{D=m,CL=k}^{**} \end{array}\right)\;$$

(10) $$y^{**}=A^{**}\cdot x^{**}-a_{j,p}^{**}$$

(11) $$RSS={y^{**}}^{{}^{\prime }}y^{**}$$

(12) $$x_{j,p,CL}=\frac{x_{CL}^{**}}{\sum_{CL=1}^{k}x_{CL}^{**}}\times 100\%$$

## 3 RESULTS AND DISCUSSION

### 3.1 Choice of parameters and clustering

**FIGURE 1:**
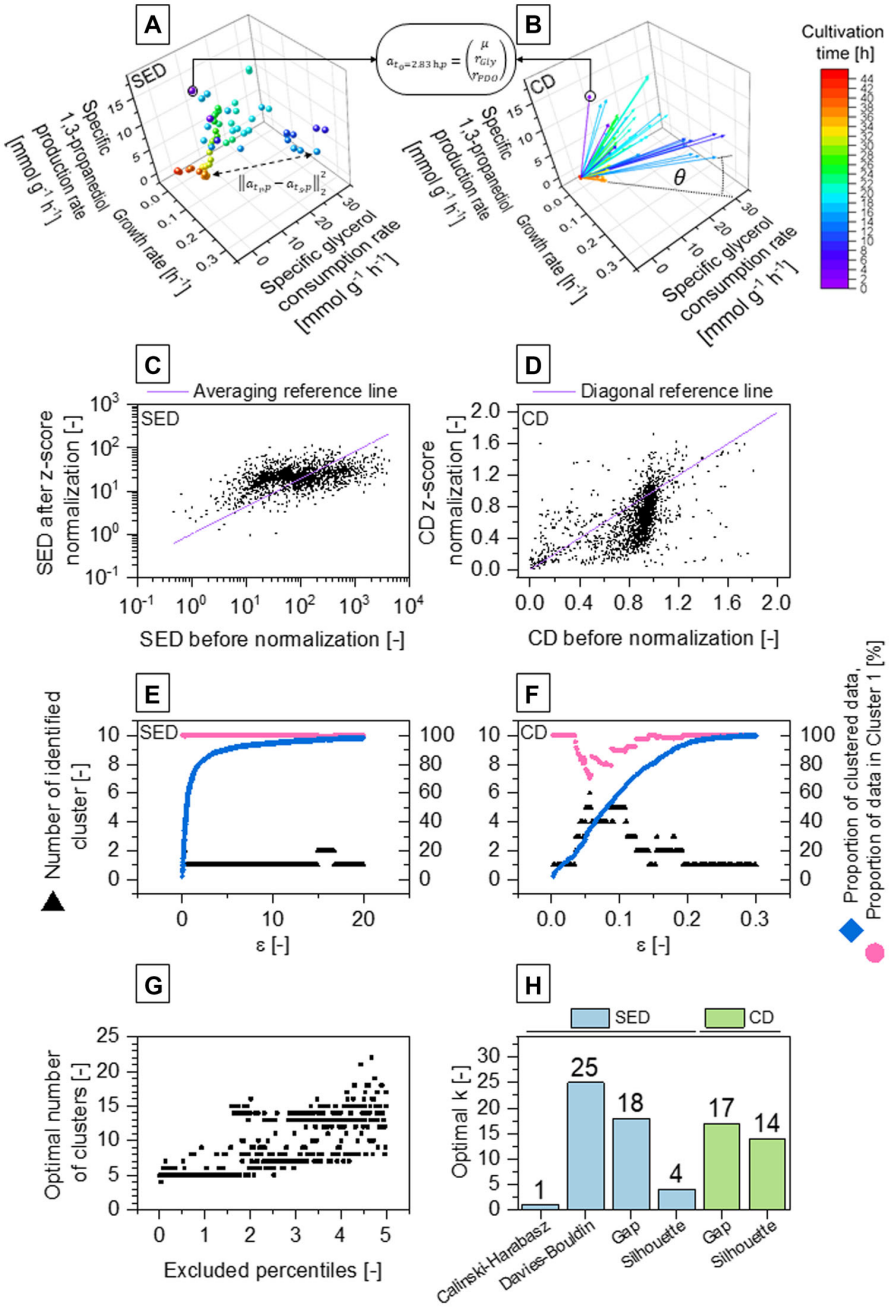
Vector display of fermentation data with utilized distance metrics, impact of *z*‐score normalization, influence of clustering parameters and computed number of optimal clusters using different criteria. (A and B) For a simplified example of three dimensions (growth rate, specific 1,3‐propanediol production rate, specific glycerol consumption rate) the vector display is shown for squared Euclidean distance (SED) and cosine distance (CD). Each point/vector $a_{t,p}$ represents the phenotypic manifestation during a cultivation experiment, which is used for clustering. For computing clusters, SED as $∥a_{t_{r},p}-a_{t_{s},p}{∥}_{2}^{2}$ between exemplary points at $t_{r}$ and $t_{s}$ or CD based on the angle θ between both vectors are used. (C and D) Valuation of distances to the mean of all data depending on the sample standard deviation via z‐score normalization for SED and CD, respectively. All distances above the reference lines represent distances that are weighted higher through consideration of sample standard deviation and vice‐versa. (E and F) Number of identified cluster and the clustering properties for Density‐based spatial clustering of applications with noise (DBSCAN) are shown for SED and CD, respectively. Proportions of clustered data (non‐noise data) and proportion of data in cluster 1 depicts the quality of DBSCAN. (G) Computed optimal number of clusters using silhouette criterion and CD metric with varying degree of outlier removal of each dimension up to 5^th^ and 95^th^ percentiles. (H) Computed optimal number of clusters using different criterions for CD and SED metric with 3^rd^ and 97^th^ percentiles of outlier removal

From 1025 sampling data consisting of concentrations (biomass, substrates and products) from different cultivation time points of 90 *C. pasteurianum* cultivations, specific rates were calculated and arranged as vectors. For clustering, a distance metric as a measure of dissimilarities must be chosen, where squared Euclidean distance (SED, Figure 1A) and cosine distance (CD, Figure 1B) were employed in this work. For a simplified case of three dimensions, SED computes the Euclidean distance between points that leads to an ellipsoidal cluster formation. Thus, phenotypic behaviors, which are described with a set of specific rates, are agglomerated within a computed range that is all similar in the scale in all dimensions. CD is based on the inner product space of two vectors, where the angle between the vectors represents the distance of two vectors as basis for CD. Thus, the “directional” traits of the vectors are clustered together independent of the scale or “length” of the vectors. In addition to distance metric, varying scalar differences and degree of scattering for different dimensions of each vector was found to introduce undesired scalar weighting. Therefore, we decided to continue with normalization of $a_{j,p}$ by taking account the sample standard deviations (4) and found the z‐score normalization to be a potent method to calculate normalized vectors $a_{j,p}^{*}$. As shown in Figure 1C,D, the calculated distances for CD and SED are transformed to lower weighted (below the reference line) or higher weighted (above the reference line) distances.

As an initial approach for clustering, we decided to apply a density‐based clustering method that can distinguish between noise or outliers and clusters. The DBSCAN clustering algorithm with its feature of finding “natural clusters” and noise detection [39] seemed promising. However, the presence of different local densities for different potential clusters that are not captured by global parameters [40] and “curse of dimensionality” for high‐dimensional datasets [41] resulted in difficulties for suitable cluster assignments. Sander et al. [42] suggested $n_{p_{min}}$corresponding to twice the number of dimensions, which conforms to 22 in this demonstrated case. Results of screening for suitable ε value employing SED and CD are shown in Figure 1E,F. When SED was used as the metric, only a maximum of two clusters were identified with proportions between 99.3% and 100% of clustered data assigned to the first cluster, which was mostly the sole cluster. For CD, up to six clusters were found (ε = 0.056) with 35.29% of data successfully assigned. Still, over 70% of assigned data were allocated to the first cluster, leading only to 10.46% of all data assorted to the remaining five clusters.

Pursuing an alternative method, we continued with k‐means as centroid‐based clustering method, which is an iterative algorithm. Data grouping is performed, in which data points in each cluster are as close to each other and as far away as possible from other clusters, resulting in most compact and well separated cluster formation [43]. As an input requirement, k as number of clusters is needed that can be predicted by several methods as described in Section 2.2. In contrast to DBSCAN, classical k‐means does not feature noise or outlier detection, inclusion of all data points without prior outlier removal will lead towards distorted clustering with imprecise centroids—especially for vectors at low biomass concentrations due to the definitions of biomass‐specific rates as in (1). Varying the percentiles for exclusion had a great influence on the clustering result: for the exemplary case of CD as distance metric and silhouette evaluation method for determination of k, tendency of increased k with increasing percentiles of excluded data points was observed (Figure 1G). For further analysis with k‐means, we continued with an exclusion of 3^rd^ and 97^th^ percentiles.

**FIGURE 2:**
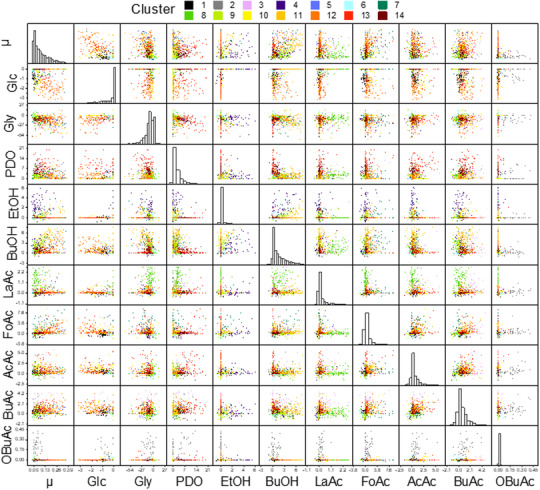
Scatter matrix for k‐means clustering of 90 *Clostridium pasteurianum* cultivation experiments. For k of 14, k‐means clustering was performed based on cosine distance metric and z‐score normalization. All 11 dimensions (growth rate (μ), specific production or consumption rates of glucose (Glc), glycerol, 1,3‐propanediol (PDO), ethanol (EtOH), butanol (BuOH), lactic acid (LaAc), formic acid (FoAc), acetic acid (AcAc), butyric acid (BuAc) and 2‐oxobutyric acid (OBuAc)) are shown in a scatterplot matrix, where the diagonal shows a histogram of each dimension as number of points with normalized scales. The units are: [h^‐1^] for growth rate, [mmol g^‐1^ h^‐1^] for other rates and [‐] for the diagonal

Overall, normalization to avoid scalar weighting, choice of distance metric depending on the intended motive and clustering parameters were the required inputs for the computing process. Independent from clustering methods, these input parameters directly and indirectly stand for number of clusters and outlier/noise elimination, and thus, accuracies of formed clusters. In this demonstrated case, the general consideration follows two contrary ideas: accurate detection of all phenotypic manifestations (e.g. SED, no outlier removal with exceptionally high k), which may also detect noise as potential clusters; or generalization of phenotypic behaviors (e.g. CD, high outlier removal rate with manageable k) with potential risk of oversimplification and overlooking in‐between phenotypic manifestations. Increase of k simultaneously increases the range and differentiation of captured cellular behavior by sacrificing manageability. Pursuing a compromise between accuracy and manageability, we continued with CD and k of 14 (based on silhouette criterion) (Figure 2). As a recommended strategy from this demonstration, estimation of k by using here employed (or alternative) criterions (in Figure 1H), gives the first indication of ranges for k. Considering accuracy vs. manageability, one can then decide and iteratively adjust specific parameters that suits the need and intention. Obviously, these parameters need to be re‐examined depending on the spectrum of metabolism as well as quality and quantity of data for alternative cases and datasets. Thus the quantitative evaluation for dataset‐based influences for phenotype analysis requires further study employing alternative datasets. Overall, under the assumption of absent additional reference phenotypes within the dataset, there will be a saturation point of input dataset leading to only marginal differences of captured clusters with increasing quantity. The quality of dataset, however, remains essential for the accuracy of desired output. In addition, with great advances and fast development of new clustering methods [44], other algorithms may be equally or better employable for such application, which require further study.

### 3.2 Cluster analysis and comparison between clusters

**FIGURE 3:**
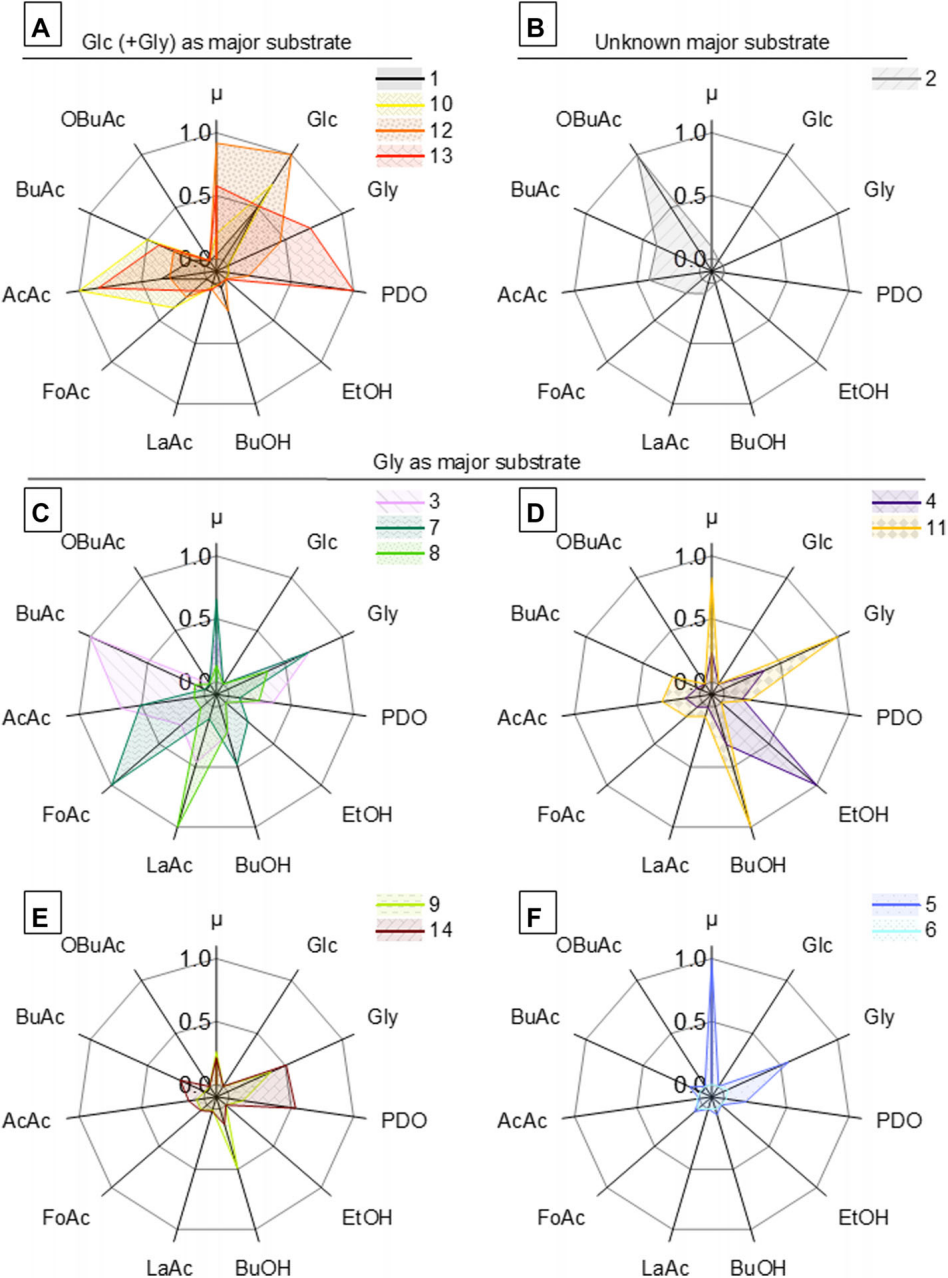
Radar charts of identified clusters of phenotypic manifestations in *C. pasteurianum*. Normalized centroids of 14 clusters of *C. pasteurianum* fermentations using cosine distance metric sorted based on the main carbon source. (A) Clusters 1 and 10 utilize glucose (Glc) as major substrate and clusters 12 and 13 utilize Glc and glycerol (Gly), while cluster 10 and 13 showed the highest acetic acid (AcAc) and 1,3‐propanediol (PDO) production rates, respectively; (B) Cluster with 2‐oxobutyric acid (OBuAc) production without apparent Glc or Gly consumption; (C–F) Clusters with Gly as major substrate, further differentiated by the product spectrum. Clusters 3, 7 and 8 (C) show highest production rates of butyric acid (BuOH), formic acid (FoAc) and lactic acid (LaAc), respectively. Highest solventogenesis of ethanol (EtOH) and butanol (BuOH) were found for clusters 4 and 11 (D), respectively. Cluster 5 and 6 (F) showed highest and lowest growth rate (μ), respectively. Clusters 9 and 14 (E) are not characteristic for a single metabolic activity

Two clusters (clusters 1 and 10) (Figure 3A) were found that utilize mainly glucose with different product spectrums. For co‐consumption of glycerol and glucose, clusters 12 and 13 were identified, while cluster 13 showed the highest 1,3‐propanediol production rate from all identified clusters. Interestingly, cluster 2 (Figure 3B) was identified as sole cluster with 2‐oxobutyric acid production and without apparent glycerol and glucose consumption. The remaining nine clusters were grouped as clusters with glycerol as sole substrate with diverse phenotypic expressions (Figure 3C‐F): from all identified clusters, the highest butyric acid (cluster 3), formic acid (cluster 7), lactic acid (cluster 8), butanol (cluster 11) and ethanol (cluster 4) production rate, as well as highest specific growth rate (cluster 5) were identified. Cluster 9 and 14 did not include any dominant production of specific metabolite and cluster 6 represents the state of no cellular growth with minimal biological activity.

The fundamental thought throughout cluster‐based assessment is that the computed centroids only represent an abstraction of the detected phenotypic manifestations, in which the correlation to reality is based on the origin of clustered data. By qualitatively comparing identified clusters to previous works with data, which was not included in this cluster analysis, high similarities were observed for multiple clusters. For instance, the highest 1,3‐propanediol production in co‐substrate rather than mono‐substrate fermentation [45] corresponds to cluster 13, where the lower production of 1,3‐propanediol on glycerol appeared at a varying spectrum of acidogenesis [27, 30, 45] as clusters 3, 8 or 14. The definition of identified clusters as “references” describes not only the potential spectra of phenotypic expression and enables enhanced comparison, but also brings additional advantages, which is further highlighted in the next sections. Comparing the clusters, the interrelation between specific production or consumption rates of each cluster can be assessed as a linked whole. In an exemplary case of butanol biosynthesis as a desired phenotypic manifestation, cluster 11 (Figure 3D) stands out with the highest specific production rate (5.23 mmol g^‐1^ h^‐1^). However, if by‐production of acids are undesired, cluster 9 (s. Figure 3E) represents a more suitable manifestation with a butanol to acid production ratio of 18.5 mol per mol of acids (vs. cluster 11 with 3.1) despite the lower specific butanol production rate of 2.72 mmol g^‐1^ h^‐1^ for cluster 9. Consequently, the overall molar glycerol‐specific yield is improved: 0.37 mol butanol per mol of glycerol (vs. cluster 11 with 0.27).

### 3.3 Conditionality of phenotypic manifestations

Through clustering, several reference phenotypes were detected enabling categorical assessment of factors and conditions according to the references. In contrast to manually analyzing influences on all possible dimensions and directions for each factor (in respect to all data sets), the categorical assessment employing clusters as references was perceived as a much simpler process. Pursuing to find potential candidates as influencing factors (potential conditionalities), features or information, which were not included for the clustering, were compared for each cluster. Since the “raw concentrations” were not directly clustered, they were defined as tags to calculate the logarithmic deviation to the total dataset as in (7): negative values represent under‐representation of specific conditions in the cluster and vice versa. Following up on the previous example, clusters 9 and 11 were compared.

**FIGURE 4:**
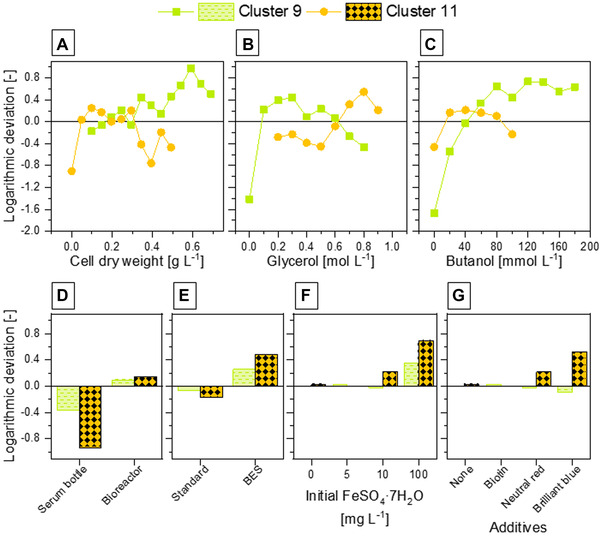
Logarithmic deviations of dynamic and general cultivations conditions (clusters 9 and 11) from total dataset. Over‐representation (logarithmic deviation >0) indicate elevated appearance of a specific cluster for a given condition in comparison to the total dataset and vice‐versa for logarithmic deviation <0. (A–C) Logarithmic deviations of cluster appearances depending on dynamic conditions (concentration ranges of cell dry weight, glycerol and butanol, respectively). Logarithmic deviations of (initial) cultivations conditions are shown for following tags: cultivation condition in pH‐uncontrolled serum bottles or bioreactors (D), cultivation employing bioelectrochemical system (BES) (E), initial iron(II) sulfate heptahydrate concentrations (F) and utilization of additives (G)

Comparing the ranges of concentrations of biomass (cell dry weight), glycerol and butanol, a trend of reciprocal representation between cluster 9 and cluster 11 was observed (Figure 4A‐C): cluster 9 was over‐representative at high biomass (≥ 0.34 g L^‐1^), lower glycerol (between 0.1 and 0.6 mol L^‐1^) and high butanol concentrations (≥ 60 mmol L^‐1^); whereby cluster 11 was over‐representative at lower biomass concentrations (between 0.05 and 0.30 g L^‐1^), higher glycerol concentrations (≥ 0.7 mol L^‐1^) and lower butanol concentrations (between 20 and 80 mmol L^‐1^). In regard of fermentation conditions, both clusters were under‐representative for pH‐uncontrolled serum bottle cultivations, and over‐representative in fermentations employing BES (Figure 4D,E). For additional additives, it appears that both clusters are over‐representative for higher concentrations of initial FeSO_4_∙7H_2_O concentrations (100 mg L^‐1^ for both clusters and 10 mg L^‐1^ only for cluster 11; Figure 4F) and cluster 11 (Figure 4G) is over‐representative for cultivations with Neutral Red and Brilliant blue addition.

This observation shows that properties that were not included directly for cluster formation were nevertheless clustered into a common grouping. However, we noticed that it is crucial to consider cause‐and‐effect relationship and bias of the input dataset. Cause‐and‐effect relationship can be highlighted with butanol concentration ranges in the demonstrated example (Figure 4C), where it is unlikely to find data near 0 mmol L^‐1^ butanol concentration, since cluster 9 and 11 are pre‐defined with high butanol production. The influence of bias from the input dataset can be shown for the initial FeSO_4_∙7H_2_O concentration, where absence of over‐ or under‐representation of cluster 9 for 10 mg L^‐1^ (Figure 4F) is simply based on missing cultivation experiments at 10 mg L^‐1^ FeSO_4_∙7H_2_O. Despite these observations, potential cultivation conditions (including dynamic conditions) were isolated as potential conditionalities. However, valuation and confirmation of potential conditionalities require additional experimental validations.

### 3.4 Cluster‐based balancing of carbons and ATP

**FIGURE 5:**
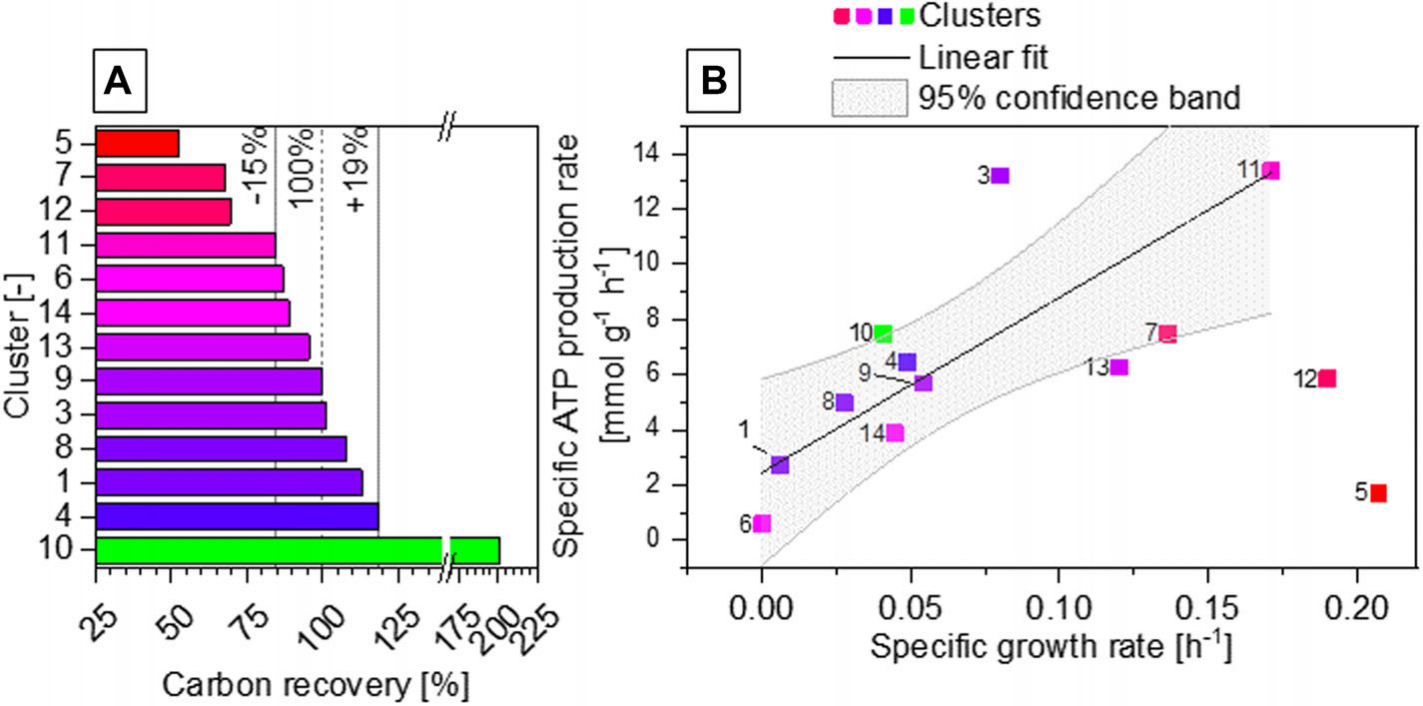
Carbon recovery and specific ATP production rates of identified clusters. (A) Carbon recoveries of identified clusters that are calculated from the characteristic sets of specific rates including theoretical carbon dioxide production rate. (B) Plot of specific ATP production rate based on substrate‐level phosphorylation against specific growth rate and linear fit excluding clusters with carbon recoveries over 19% discrepancy. Cluster 2 constitutes an exception, since no identified substrate uptake was found disabling calculation of carbon recovery and specific ATP production rate

To demonstrate the practicability of utilizing clusters as reference manifestations and to validate the characteristics of detected clusters, we continued to analyze the carbon recovery $R_{C}$ (13) considering theoretical CO_2_ production rate (14) based on the product formation (rate of decarboxylation of pyruvate), where$\;n_{C}$ equates to number of carbons per mol of the compound (Table 1). With the exception of cluster 2 (unidentified carbon intake), other clusters were found to represent phenotypic expression with carbon recoveries between 53% and 201% (Figure 5A). In general, carbon recoveries lower than 100% can be interpreted as metabolic products (e.g. primary metabolites) missing from the analysis. Carbon recoveries over 100% indicate potential substrates not factored during measurement (e.g. complex compounds, such as yeast extract). Despite the origin of the datasets that includes the whole range of dynamic cellular behavior, the majority of clusters showed recoveries close to 100%. The exceptions are cluster 5, 7 and 12 that showed recoveries between 53% and 70%, while cluster 10 showed over 200% carbon recovery. Thus, we continued with further analysis based on the assumption that discrepancy over 19% (from 100%) is generated from here neglected cellular metabolism beyond the range of basal cellular dynamics.

(13) $$R_{C}\left(CL\right)={\left.\frac{n_{C_{BM}}\frac{\zeta_{\mu }}{M_{BM}}+\sum_{D=4}^{m}n_{C_{D}}\zeta_{D}+r_{CO_{2}}}{n_{C_{Glc}}\zeta_{Glc}+n_{C_{Gly}}\zeta_{Gly}}\right|}_{CL}$$

(14) $$\begin{array}{c} \begin{array}{c} r_{CO_{2}}\left(CL\right)={\left.\left(\zeta_{EtOH}+\zeta_{AcAc}+2\left(\zeta_{BuOH}+\zeta_{BuAc}\right)-\zeta_{FoAc}\right)\right|}_{CL} \end{array} \end{array}$$

**TABLE 1:** Dimensions of cluster analysis and corresponding definition of substrates and products with their properties used for calculations in this work

Dimension (D)	Compound	Abbreviation	Molar mass ($M_{D}$)	Chemical formula	Number of carbons ($n_{C_{D}}$)	Stoichiometric ATP yield ($s_{ATP/D}$)
[‐]	[‐]	[‐]	[g mol^‐1^]	[‐]	[mol mol^‐1^]	[mol mol^‐1^]
1	Biomass or specific growth rate	BMμ	101.1	C_4_H_7_O_2_N (Biebl [27])	4	$\frac{M_{BM}}{Y_{BM/ATP}\;}$
2	Glucose	Glc	180.2	C_6_H_12_O_6_	6	–
3	Glycerol	Gly	92.09	C_3_H_8_O_3_	3	–
4	1,3‐Propanediol	PDO	76.09	C_3_H_8_O_2_	3	0
5	Ethanol	EtOH	46.07	C_2_H_6_O	2	1
6	Butanol	BuOH	74.12	C₄H_10_O	4	2
7	Lactic acid	LaAc	90.08	C_3_H_6_O_3_	3	1
8	Formic acid	FoAc	46.03	CH₂O₂	1	0
9	Acetic acid	AcAc	60.05	C_2_H_4_O_2_	2	2
10	Butyric acid	BuAc	88.11	C_4_H_8_O_2_	4	3
11	2‐Oxobutyric acid	OBuAc	102.1	C_4_H_6_O_3_	4	–

For analysis and balancing of fermentation‐related data from *C. pasteurianum*, one of the major important unknown variables remains the energy metabolism on ATP‐level, which was approached from the perspective of “clusters as reference phenotypes”: based on the assumption of ATP biosynthesis solely from substrate‐level phosphorylation, (15) was assumed, where $s_{ATP/D}$ equates stoichiometric molar ATP yield [mol mol^−1^] and $Y_{BM/ATP}$ ATP‐specific biomass yield [mol g^−1^] (Table 1). The demand for “maintenance” metabolism was simplified as $q_{ATP}^{m}$. This balancing neither includes an extensive kinetic model nor energetic considerations based on inhibition terms or on substrate availability, which leads to additional effects, such as “energy spilling” [46]. Since the origin of data, which was used for unsupervised learning, cover all dynamic phases of bacterial growth, we pursued a more generalized calculation based on the known and expected range of metabolic spectrum. Hence, with the exclusion of clusters beyond 19% deviation from full carbon recovery, an unweighted linear regression (Figure 5B) was made to assess the unknown variables. The computed $Y_{BM/ATP}$ was (10.18 ± 2.9) g mol^−1^ that surprisingly well matches to the reported and often used yield of 10.1 to 10.5 g mol^‐1^ for 1,3‐propanediol fermentations [47, 48, 49] with $q_{ATP}^{m}$ of (2.46 ± 1.43) mmol g^‐1^ h^‐1^. However, the presence of “carbon recovery outliers” as clusters 5, 7, 10, and 12 or clusters 3 and 13 outside of the 95% confidence band of the linear fit clearly indicate presence of “unidentified” part of the energy metabolism. The neglected or unidentified energy metabolism can be repeatedly observed in cultivation manifestations of *C*. *pasteurianum* as described by 6 of in total 14 clusters, which does not coincide with the generic fermentation behavior. Thus, (15) appears only to be limited for specific cellular manifestations and additional factors are present, which strongly affects the energetic balance (e.g. substrate availability, overflow metabolism). Further, the degree of such influence onto energy metabolism for each reference phenotypic manifestation can be hereby assessed. Thus, by categorizing reference phenotypes to assumed or expected relationships (here as balancing), validity of such assumptions can be assessed in an abstracted manner, so that all datasets do not need to be individually analyzed and compared.

(15) $$\sum_{D=4}^{m}s_{ATP/D}\;\zeta_{D}=\frac{1}{Y_{BM/ATP}}\mu +q_{ATP}^{m}$$

### 3.5 Cluster‐based approximation of cellular behavior

**FIGURE 6:**
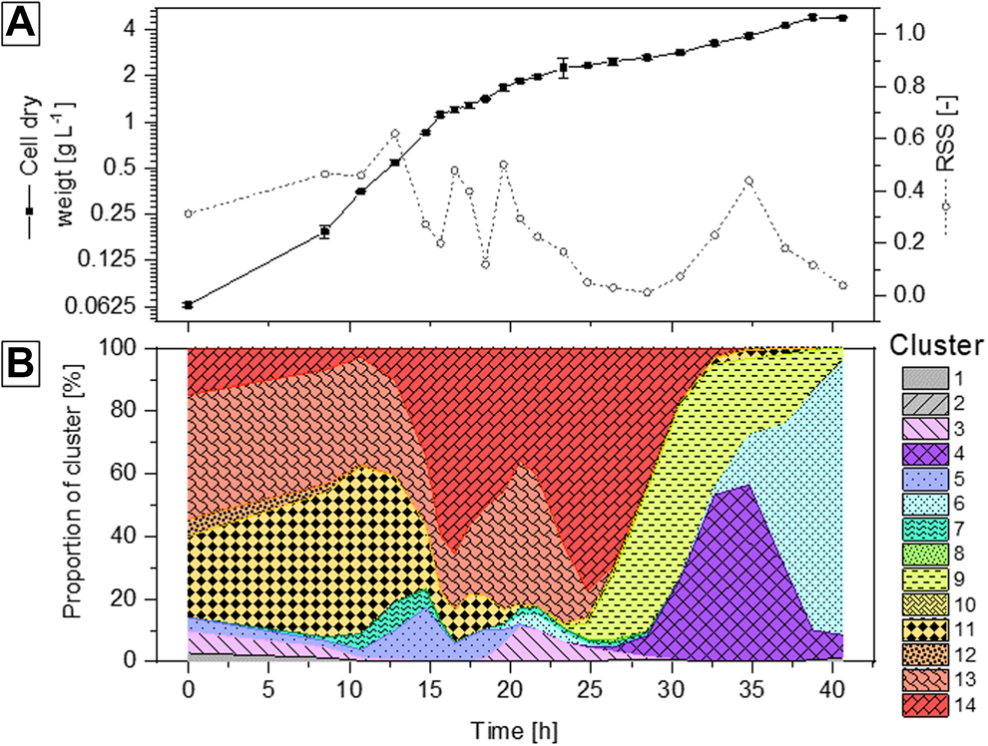
Superposition‐based approximation of batch cultivation of *C. pasteurianum*. (A) Time course of cell dry weight of the batch fermentation and residual sum of squares (RSS) of the non‐negative least square fitting of cluster‐based approximation. (B) Proportions of identified clusters as superposition‐based non‐negative least square fitting of all identified 14 clusters that describe dynamic states of phenotypic manifestation as summed composition

If clusters can be formed that constitutes reference phenotypic manifestations, analogousness of detected phenotype of interest to a specific cluster (or combination of clusters) may be used for comparison or description of cellular behavior. Based on the superposition‐principle, the idea of describing a phenotypic state as proportions of reference clusters was applied (e.g. phenotype of interest equals 60% reference cluster 1 and 40% reference cluster 2; rather than listing of all specific rates). For its demonstration, an internal validation was performed for a batch‐fermentation on glycerol utilizing the *C. pasteurianum* R525 strain, while the quality of the approximation (non‐negative least square fitting) is described as residual sum of squares (RSS) (Figure 6A). Shortly after initiation of the fermentation, approx. 30% to 40% of cluster 13 (high acetic acid and 1,3‐propanediol production) and 11 (high butanol production) represent the phenotypic behavior. Comparing these two clusters, the only common feature of clusters 11 and cluster 13 are the relatively high growth rates of 0.171 and 0.120 h^‐1^, respectively. The presence of two cluster‐specific manifestations during the lag phase (and transition to the exponential growth phase) potentially indicates cellular adaptation as diverse phenotypes. Then, cluster 14 becomes with the highest representative cluster (up to 77%) during the growth phase (starting at approx. 15 h) with relatively dominant 1,3‐propanediol production rate of 5.22 mmol g^‐1^ h^‐1^ and minor acid production rate of 0.84 mmol g^‐1^ h^‐1^. Interestingly, nearing the stationary phase, three temporary transitions are to be seen: starting with cluster 9 (2.72 mmol g^‐1^ h^‐1^ butanol production rate), followed by cluster 4 (2.23 mmol g^‐1^ h^‐1^ ethanol production rate) and cluster 6 (almost no biological activity).

Such simplified description does not provide any benefits, when individual cultivation experiments are considered. However, for established production processes, where the process is repeatedly running and the desired phenotypic manifestation is clearly defined (or ranked), such reference‐based description may constitute a tool that indicates the process state as an entirety of phenotypic manifestations rather than relying on individual parameters (e.g. production rate of product).

## 4 CONCLUDING REMARKS

The demonstrated method of unsupervised learning for identification of clusters as reference manifestations enables simplified processing and comprehensive comparison of phenotypic manifestation from multiple series of fermentation data within seconds. Also, by abstracting excessive number of “data points” as representative centroids, streamlined analysis is enabled (1025 sampling points → 14 clusters) as demonstrated by few examples of cluster‐based analysis.

While the automated process of learning to abstract generic phenotypic behavior was demonstrated successfully for *C. pasteurianum*, the required inputs and parameters needed individual consideration for transfer of this methods to alternative dataset (other strains or types of data), where the operator needs to readjust clustering parameters to obtain desired clustering accuracy and manageability.

## NOMENCLATURE

**Table:** 

$A^{**}$	[‐]	Matrix of all [0,1]‐rescaled centroids of all clusters
M	[g mol^−1^]	Molar mass
$R_{C}$	[%]	Carbon recovery
RSS	[‐]	Residual sum of squares
S	[various]	Sample standard deviation
$Y_{BM/ATP}$	[g mol^−1^]	ATP‐specific biomass yield coefficient
a	[various]	Vector of specific rates (μ and $r_{D}$) as phenotypic manifestation
c	[g L^−1^ or mmol L^−1^]	Concentration
ce	[‐]	Cultivation experiment
$d_{CD}$	[‐]	Cosine distance as measure of dissimilarity

**Table:** 

$d_{SED}$	[various]	Squared Euclidean distance as measure of dissimilarity
k	[‐]	Number of clusters
$n_{C}$	[‐]	Number of carbon
$n_{CL,\alpha_{j,p}}$	[‐]	Number of vectors ($a_{j,p}$) assigned to a specific cluster CL
$n_{p_{min}}$	[‐]	Minimum number of neighbors for a core point used for DBSCAN
$q_{ATP}^{m}$	[mmol g^‐1^ h ^‐1^]	Biomass‐specific ATP consumption rate for cellular maintenance
r	[mmol g^‐1^ h ^‐1^]	Biomass‐specific rate
$s_{ATP/D}$	[‐]	Stoichiometric factors for ATP recovery via substrate‐level phosphorylation for biosynthesis of compound in D
t	[h]	Time
x	[‐]	Portions of cluster for superposition‐based approximation
y	[‐]	Residual term of the non‐negative least squares fitting problem
z	[‐]	Z‐score normalized specific rates (μ and $r_{D}$)
Greek symbols		
$\delta_{cond}$	[‐]	Logarithmic difference of a specific cluster to the sample population incl. all clusters
ε	[‐]	Scalar for neighborhood search radius used for DBSCAN
ζ	[various]	Centroid of a cluster
θ	[‐]	Angle between two vectors
μ	[h^−1^]	Specific growth rate
Indices		
∗	[‐]	Z‐score normalized value
∗∗	[‐]	To [0,1]‐rescaled value
CL	[‐]	Cluster [1, k]
D	[‐]	Dimensions of phenotypic manifestations (biomass, substrates, metabolites) [1, m]
i	[‐]	Indices for sampling points [1, n]
j	[‐]	Indices for PCHIP‐interpolated points [1, $j_{max}$]
p	[‐]	Indices for cultivation experiment [1, $p_{max}$]

Comparable to other machine learning methods, the quality and quantity of the original data set influences greatly the results. In this regard, customized “filter” for raw data, as well as additional weighting of specifically required parameters or alternative algorithms, can be additionally employed to the presented method. Nevertheless, the usage and processing of “raw” concentration data is possible as it was demonstrated in this work. For its full exploitation and accentuation of its perks and identification of other limitations, application and comparison of clustering based on alternative data sets is necessary. Clearly purposed data (e.g. cultivation data of industrial fermentation) would also be an opportune approach for a qualitative assessment of its avail.

## CONFLICT OF INTEREST

The authors have declared no conflict of interests.

## Supporting information

SUPPORTING INFORMATION

## Data Availability

Data sets and MATLAB scripts related to this work can be found at Mendeley Data repository: Hong, Yaeseong (2021), “Phenotype analysis of cultivation processes via unsupervised machine learning: demonstration for Clostridium pasteurianum”, Mendeley Data, V1, doi: 10.17632/twcpbbt3rx.1
