# Supplementary material for: Phenotype analysis of cultivation processes via unsupervised machine learning: Demonstration for Clostridium pasteurianum
Source: Eng Life Sci. 2021 Dec 10;22(2):85–99. doi: 10.1002/elsc.202100114 (PMC8811730; doi:10.1002/elsc.202100114)
Supplement: Supplementary file 1 — SUPPORTING INFORMATION [file ELSC-22-85-s001.pdf]

Supporting Information

Phenotype analysis of cultivation processes via unsupervised machine learning: demonstration for *Clostridium pasteurianum*

Yaeseong Hong[1,+], Tom Nguyen[1,+], Philipp Arbter[1], Tyll Utesch[1], An-Ping Zeng[1]

[1] Hamburg University of Technology, TUHH, Institute of Bioprocess and Biosystems Engineering, Hamburg, Germany

[+] These authors contributed equally

Correspondence: Prof. An-Ping Zeng (aze@tuhh.de). Hamburg University of Technology, TUHH, Institute of Bioprocess and Biosystems Engineering, Hamburg, Germany.

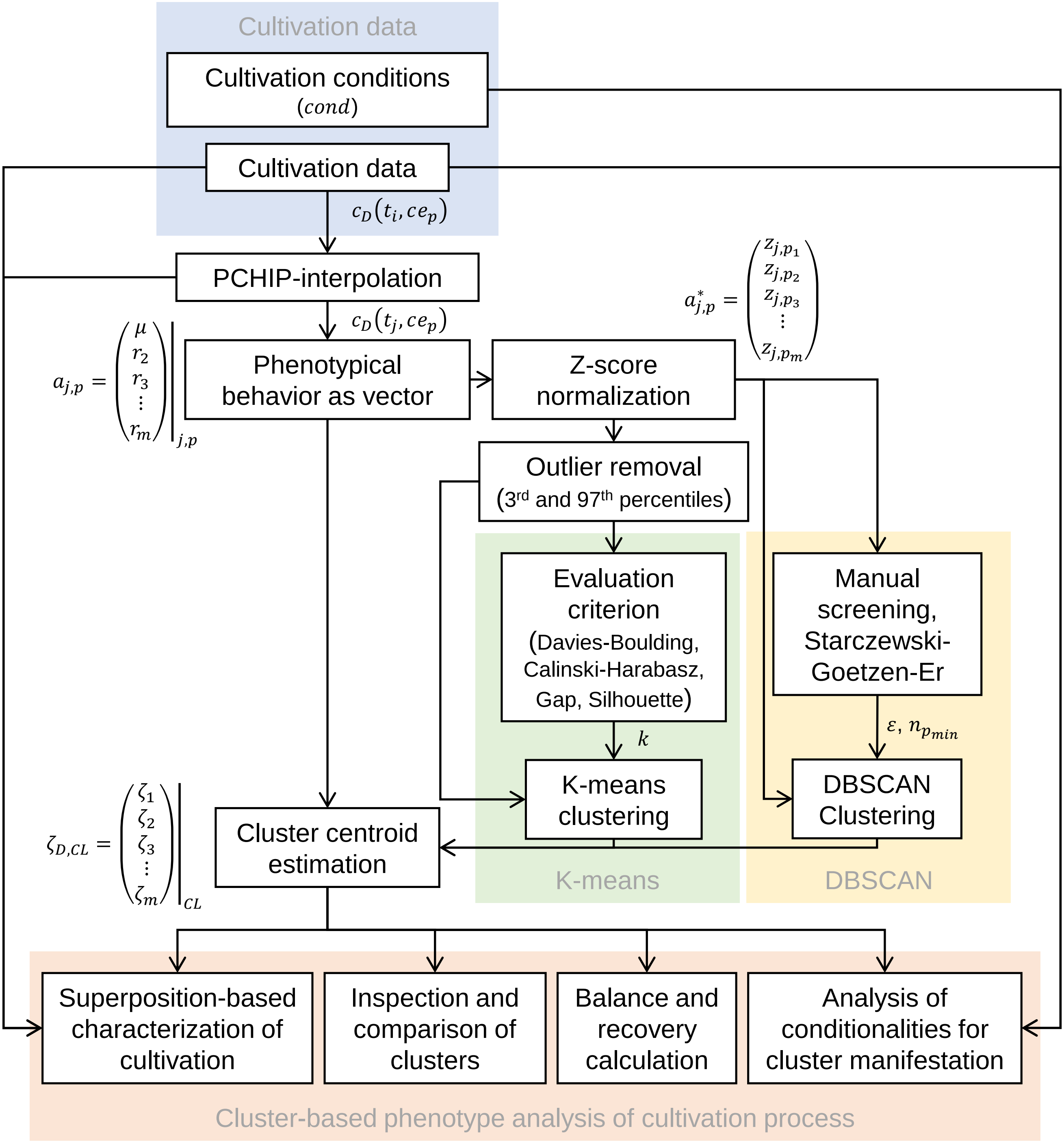

Supporting Information 1:

Cluster-based phenotype analysis used in this work.

Calculation order of cluster-based phenotype analysis as flowchart: cultivation data ($c_D(t_i, ce_p)$) are PCHIP-interpolated ($c_D(t_j, ce_p)$) for the calculation of the vector of specific rates ($a_{j,p}$). After z-score normalization (resulting in $a^*_{j,p}$), k-means clustering or Density-based spatial clustering of applications with noise (DBSCAN) is performed that results in cluster assignments of $a^*_{j,p}$, which is transferred to $a_{j,p}$ for the estimation of cluster centroids ($\zeta_{D,CL}$), that depicture typical and distinct cultivation behaviors. After capture of centroids, various analyses can be performed that are demonstrated in this work.

Supporting Information 2:

Clostridium pasteurianum strains used in this study.

***C. pasteurianum* strains**	**Description**	**Source**
DSM525	Type strain from DSMZ-German Collection of Microorganisms and Cell Cultures GmbH, Braunschweig, Germany	DSMZ
R525	Selected *C. pasteurianum* DSM 525 with increased transformation efficiency	a)
R525 PC	Overexpression of pyruvate carboxylase (pyc)	b)
R525 dhaBKO	Deletion of glycerol dehydratase (dhaB)	a)
R525 GCSY1	Overexpression of glycine synthase proteins of *Gottschalkia acidurici* DSM 604 (gcvT, gcvH, $gcvP_A$, $gcvP_B$, lpd)	c)
TK	Isolated *C. pasteurianum* strain from environmental sample	d)

[a)] Schmitz, R., Sabra, W., Arbter, P., Hong, Y. et al., Improved electrocompetence and metabolic engineering of Clostridium pasteurianum reveals a new regulation pattern of glycerol fermentation. *Engineering in Life Sciences* 2019, 19, 412–422.

[b)] Schmitz, R., *Metabolic engineering von Clostridium pasteurianum zur Optimierung der Biobutanolproduktion*, 2018.

[c)] Hong, Y., Arbter, P., Wang, W., Rojas, L. N. et al., Introduction of glycine synthase enables uptake of exogenous formate and strongly impacts the metabolism in Clostridium pasteurianum. *Biotechnology and bioengineering* 2021, *118*, 1366–1380.

[d)] Kaeding, T., DaLuz, J., Kube, J., Zeng, A.-P., Integrated study of fermentation and downstream processing in a miniplant significantly improved the microbial 1,3-propanediol production from raw glycerol. *Bioprocess and biosystems engineering* 2015, *38*, 575–586
